# Down-Regulation of LOC645166 in T Cells of Ankylosing Spondylitis Patients Promotes the NF-κB Signaling *via* Decreasingly Blocking Recruitment of the IKK Complex to K63-Linked Polyubiquitin Chains

**DOI:** 10.3389/fimmu.2021.591706

**Published:** 2021-02-25

**Authors:** Hui-Chun Yu, Kuang-Yung Huang, Ming-Chi Lu, Hsien-Yu Huang Tseng, Su-Qin Liu, Ning-Sheng Lai, Hsien-Bin Huang

**Affiliations:** ^1^ Department of Medical Research, Dalin Tzu Chi Hospital, Buddhist Tzu Chi Medical Foundation, Chiayi, Taiwan; ^2^ Division of Allergy, Immunology and Rheumatology, Department of Medicine, Dalin Tzu Chi Hospital, Buddhist Tzu Chi Medical Foundation, Chiayi, Taiwan; ^3^ School of Medicine, Tzu Chi University, Hualien, Taiwan; ^4^ Department of Biomedical Sciences, National Chung Cheng University, Chiayi, Taiwan

**Keywords:** ankylosing spondylitis, long noncoding RNA, LOC645166, polyubiquitin, NF- kappa B

## Abstract

Ankylosing spondylitis (AS) is a chronic inflammatory disease that mainly affects the spine. AS is highly associated with the expression of HLA-B27. Up to 95% AS patients are HLA-B27-positive. However, only 1%–2% of the HLA-B27-positive carriers suffer from AS, implying that other factors may also govern the development of AS. Long non-coding RNAs (lncRNAs) can regulate the immune response *via* their binding proteins. In the present study, we have identified that the levels of lncRNA, LOC645166, in T cells of AS patients were reduced. Overexpression of LOC645166 in Jurkat cells down-regulated the IL-23p19 expression and suppressed the JAK2/STAT3 signaling in response to stimulation by phorbol 12-myristate 13-acetate. Suppression of STAT3 activation by LOC645166 was also observed when Jurkat cells or T cells of AS patient were treated with anti-CD3/CD28 antibodies. In order to explore the role of LOC645166 in the pathogenesis of AS, RNA pull-down assay plus proteomic approach and western blotting were performed and identified that LOC645166 prefers binding the K63-linked polyubiquitin chains. LOC645166 can suppress recruitment of the IKK complex to K63-linked polyubiquitin chains and diminish IKK2 activation, leading to down-regulation of NF-κB activation. Down-regulation of LOC645166 expression in T cells of AS patients up-regulates NF-kB activation *via* decreasingly impeding recruitment of the IKK complex to K63-linked polyubiquitin chains, allowing AS patients to exhibit more sensitivity to stimulation by the proinflammatory cytokines or by TLR ligands.

## Introduction

Ankylosing spondylitis (AS) is a chronic systemic inflammatory disease. The pathology of AS has a strong influence on the enthuses where tendons, ligaments, and capsules are integrated into the bone. An inflammatory reaction in the spine is always observed in the early stage of AS symptoms. Some patients will develop ankylosis and syndesmophytes with time (1–4). The expression of HLA-B27 is highly involved in the pathogenesis of AS (5, 6). Up to 95% AS patients are HLA-B27-positive. However, only around 1%–2% of all HLA-B27-positive persons suffer from AS, implying that other genetic and environmental factors may also be involved in the development of AS (1–4). To identify the effects of each possible factor will allow us to better understand the progression of AS pathogenesis and discover a potential therapy for AS.

Several lines of evidence obtained from recent studies have demonstrated that long non-coding RNAs (lncRNAs) play the important roles in regulation of immune responses (7). The locations of many immune-related lcnRNAs are adjacent to, or partly overlapping, the 5’ end or 3’ end of protein-coding genes involved in immune response (8, 9). These immune-related lncRNAs can serve as the cis-acting factors or trans-acting factors to modulate the expression of the adjacent protein-coding genes, bringing the distal enhancer regions into vicinity with the promoter regions (8–11) or providing the domains for protein bindings or base-pairing with RNA or DNA sequences (12–17). As a result, lncRNAs can regulate the innate immune response as well as T cell activation, development, and differentiation (9).

NF-κB activation plays a critical role in inflammatory reaction and immune responses through regulating the expression of pro-inflammatory cytokines (18, 19). Recent studies indicated that activation of NF-κB requires the degradable and non-degradable types of polyubiquitin conjugation systems (20–22). NF-κB is inactivated by association with the inhibitor, IκBs, allowing the inactive complex retained in the cytoplasm. In the canonical activation pathway, the IKK (IκB kinase) complex, consisting of IKK1, IKK2, and NEMO, is activated in response to stimulation by the inflammatory cytokines or Toll-like receptor (TLR) ligands (20, 23–26). The activated IKK catalyzes the phosphorylation on the specific Ser residues of IκBs, allowing the specific ubiquitin ligase complex, SCF^βTrCPs^, to recognize the IKK-phosphorylated IκBs and to trigger the K48-linked polyubiquitination reaction on the phosphorylated IκBs. The IKK-phosphorylated IκBs conjugated with the K48-linked polyubiquitin chain are in turn targeted to the proteasome for degradation (27). Without association with IκBs, NF-κB enters the nucleus and regulates the transcription of target genes (18, 19).

The non-degradable forms of polyubiquitin chains with K63-, linear-, or K11- linkages synthesized by a complicatedly posttranslational modifications are also involved in activation of IKK (20, 28–33). The inflammatory cytokines or ligands of TLR bind to their receptors and then trigger the K63 or K11-linked polyubiquitin chain conjugated onto the specific subunit of their receptors, or polyubiquitin linear chain conjugated onto NEMO of IKK complex (34–40). These non-degradable polyubiquitin chains provide the binding sites for NEMO of another IKK complex. After multimerization of IKK complex on the non-degradable polyubiquitin chains, IKK2 dimerizes and is phosphorylated by trans-autophosphorylation, leading to activating IKK complex and in turn phosphorylating IκBs for K48-linked polyubiquitination and degradation (28). In addition, the K63-linked polyubiquitin chain can also provide the binding sites for TAB2/3 of TAK1-TAB1-TAB2/3 complex (28). Thereby, TAK1 phosphorylates IKK2 to activate IKK complex that has been recruited on the polyubiquitin chain (41).

In this study, we have systematically analyzed the widespread changes of lncRNA expression in T cells of AS patients and healthy controls by using microarrays plus quantitative real-time PCR and identified that the levels of LOC645166 were significantly down-regulated in T cells of AS patients. RNA pull-down assay demonstrated that LOC645166 prefers to associate with K63-linked polyubiquitin chains and suppresses recruitment of IKK complex to K63-linked polyubiquitin chains. Overexpression of LOC645166 can suppress the NF-κB activation, as evidenced by reduction of both IKK2 and IκB phosphorylations as well as by attenuation of the p50 transportation into the nucleus in response to TNF-α treatment.

## Materials and Methods

### Materials

Tris base, dithiothreitol, acrylamide, N,N’-methylenebisacrylamide, sodium dodecyl sulfate (SDS), glycine, ammonium persulfate, NP-40, TEMED, and phorbol 12-myristate 13-acetate (PMA) were purchased from Sigma-Aldrich (St. Louis, MO, USA). Anti-IKK2, anti-phospho-IKK2 (Ser180), anti-STAT3, anti-phospho-STAT3 (Tyr705), anti-JAK2, anti-phospho-JAK2 (Tyr1007/1008), anti-ubiquitin, anti-K63-linkage specific polyubiquitin, anti-lamin A/C, anti-actin, anti-p50, anti-IκBα and anti-phospho-IκBα (Ser32) antibodies were purchased from Cell Signaling Technology (Danvers, MA, USA). Tetraubiquitin, K48-linked and K63-linked polyubiquitin chains were ordered from Boston Biochem Inc. (Cambridge, MA, USA). The streptavidin-coupled Dynabeads were purchased from Invitrogen (Carlsbad, CA, USA). Anti-CD3 and anti-CD28 antibodies were purchased from BioLegend Inc. (San Diego, CA, USA). The recombinant TNF-α was ordered from Cell Guidance Systems (St. Louis, MO, USA).

### Microarray Analysis

Human PBMCs from three AS patients and three healthy controls were isolated, respectively, following the methods as described (42). Isolation of T cells from each of the individual human PBMCs were carried out by using anti-human CD3 magnetic beads following the instruction provided by the kit of IMag Cell Separation System (BD Bioscience, Franklin Lakes, USA). Total RNAs of T cells were extracted by using QIAamp RNA Blood Mini kit (QIAGEN, GmbH, Germany). The levels of lncRNAs were analyzed by using Agilent SurePrint Microarray (Agilent Technologies, Santa Clara, CA, USA), following the methods as described by manufacturer. 0.2 μg of total RNAs was amplified and labeled with Cy3 by using a Low Input Quick-Amp Labeling kit (Agilent Technologies). 0.6 μg of Cy3-labled cRNAs was fragmented to an average size of about 50–100 nucleotides by incubation with fragmentation buffer at 60°C for 30 min and hybridized to Agilent SurePrint Microarray (G4851C) at 65°C for 17 h. After washing and drying by nitrogen gun blowing, microarrays were scanned with an Agilent microarray scanner at 535 nm for Cy3 and analyzed by Feature extraction10.5.1.1 software.

### Analysis of the lncRNAs by Quantitative RT-PCR

The expression levels of targeted lncRNAs in T cells of AS patients (n = 30) or healthy controls (n = 20) were analyzed by quantitative RT-PCR using the primers shown in **Supplementary Table I**. Relative expression levels of mRNA were defined by the following equation: (40 – threshold cycle [Ct] after being adjusted by the expression of 18S rRNA).

### The Effects of LOC645166 Overexpression in Jurkat Cells on the mRNA Expression of Proinflammatory Cytokines

The full-length of lncRNA LOC645166 was cloned by RT-PCR using the primers, 5’-ACA GCG CGG GCG CAG GCG-3’/5’-TTT TTT CCA TGG GAA ATG AAG CG-3’. The resulting product was digested by Xho I/EcoR I and subcloned into the plasmid, pcDNA3.1/mys-His(-)A, to generate the plasmid, pcDNA3.1-LOC645166. Jurkat cells (1.5 x 10^6^ cells) were transfected with pcDNA3.1-LOC645166 (1 µg) or with pcDNA3.1 (1 µg) by using electroporation. The transfected cells were maintained in RPMI-1640 medium with 10% fetal calf serum and PMA (10 ng/ml) at 37°C with 5% CO_2_. After 24 h, cells were harvested by centrifugation. The total RNAs of cells were isolated by using the QIAamp RNA Blood Mini kit (QIAGEN). IFN-γ, TNF-α, IL-6, IL-17A, or IL-23 mRNA was amplified by real-time PCR using a One Step SYBR Ex Taq qRT-PCR kit (TaKaRa, Shiga, Japan) (42). Relative expression levels of mRNA were defined as the above-mentioned method.

### LOC645166 Pull-Down Assay

The biotin-labeled LOC645166 was *in vitro* transcribed from pcDNA3.1-LOC645166 by using T7 RNA polymerase (Ambion Inc., Austin, TX, USA) in the presence of the kit for RNA biotin-labeling (Biotin RNA Labeling Mix Roche, Basel, Switzerland). For the control, the biotin-labeled RNAs were transcribed from pcDNA3.1. The resulting product was purified with the RNeasy Mini Kit (Qiagen). The nuclear proteins were extracted by the methods as described (43). For pull-down of nuclear proteins, biotinylated LOC645166 (1 µg) was mixed with 1.5 mg of nuclear extract resuspended in the IP buffer (150 mM NaCl, 20 mM Tris-HCl (pH 7.4), 1 mM EDTA, 0.5% Triton X-100 with protease inhibitors (Millipore, Burlington, MA, USA) and RNaseOUT) supplemented with 0.2 mg/ml heparin, 0.2 mg/ml yeast tRNA, and 1 mM dithiothreitol (44). All components were allowed to stand at 4°C for 2 h, followed by addition of the washed streptavidin-coupled Dynabeads (60 µl). After incubation at 4°C for 2 h, the precipitates were washed four times with the IP buffer and collected by centrifugation (760 x g) at 4°C for 2 min. The precipitated products were subjected to analysis by shotgun proteomics. To characterize the preferred binding, biotin-labeled LOC645166 (0.5 µg) dissolved in 1ml of IP buffer was incubated with 1 µg of K63-linked (or K48- or linear-linked) polyubiquitin chains. All components were incubated at 4°C for 2 h. The washed streptavidin-coupled Dynabeads (60 µl) were added to each binding reaction and further incubated at 4 °C for 1 h. Beads were washed briefly four times with IP buffer and subjected to analysis by western blotting, probed for ubiquitin.

### Co-Immunoprecipitation by Anti-IKK2 Antibody

Jurkat cells (1.5 x10^6^ cells) were transfected with pcDNA3.1-LOC645166 (1 µg) or with pcDNA3.1 (1 µg) as described by the abovementioned method. The transfected cells were maintained in RPMI-1640 medium with 10% fetal calf serum at 37°C with 5% CO_2_ for 24 h, followed by treatment with TNF-α (20 ng/ml) for 6 h. Cells were ruptured by the extraction buffer (50mM Tris-HCl, 0.2M NaCl, 1%NP40). After centrifugation at 13,000 g for 10 min, the extracted proteins in the supernatant were isolated. Co-immunoprecipitation was carried out by using protein G immunoprecipitation kit (Sigma-Aldrich). For immunoprecipitation, all components containing the extracted proteins (400 µg) and anti-IKK2 antibody (6 µl) in 600 µl of the IP buffer were incubated at 4°C for 2 h, followed by addition of protein G-conjugated agarose (60 µl) and incubation at 4°C overnight. The co-immunoprecipitated proteins were analyzed by western blotting, probed by anti- K63-linked specific polyubiquitin antibody or by anti-IKK2 antibody.

### Western Blotting

Jurkat cells were transfected with pcDNA3.1-LOC645166 or pcDNA3.1, following the above-mentioned method. Cells were maintained in RPMI-1640 medium with 10% fetal calf serum at 37°C with 5% CO_2_ and treated with PMA (10 ng/ml) or with anti-CD3 (1 µg)/anti-CD28 (1 µg) antibodies for 24 h. The transfected cells treated with TNF-α (20 ng/ml) followed the above-mentioned method. Then, cells were harvested by centrifugation, resuspended in 100 µl of 1% SDS and analyzed by western blotting, probed for actin, IκBs, phospho-IκBs, IKK2, phospho-IKK2, STAT3, phospho-STAT3, JAK2, or phospho-JAK2.

### Analysis of p50 Nuclear Localization

Jurkat cells (1.5 × 10^6^ cells) were transfected with pcDNA3.1 (1 µg) or pcDNA3.1-LOC645166 (1 µg) by using electroporation and cultured in RPMI-1640 medium with 10% fetal calf serum and 5% CO_2_ at 37°C for one day, followed by treatment with TNF-α (20 ng/ml) for 6 h. Isolation of the nuclear proteins followed the methods as described (43). The nuclear proteins was resuspended in 1% SDS and analyzed by western blotting, probed for lamin A/C and p50.

### Ethics Statement

Patients defined followed the modified New York criteria (45). Patient enrollments into the study took place from July, 2015 to June, 2018 in Buddhist Dalin Tzu-Chi General Hospital, Chia-Yi, Taiwan. All participants have signed an informed consent form approved by the Institutional Review Board and Ethics Committee (IRBEC) of Buddhist Dalin Tzu-Chi General Hospital. The protocol for isolation of human T cells from PBMCs of AS patients and healthy controls has been reviewed and approved by IRBEC. The IRBEC approval number is B10402024.

### Statistical Analysis

Data are presented as means ± SD. Statistical significance was analyzed by the Mann–Whitney U test. P-values < 0.05 were considered statistically significant.

## Results

### LOC645166 Is Down-Regulated in AS Patients

**Figure 1:**
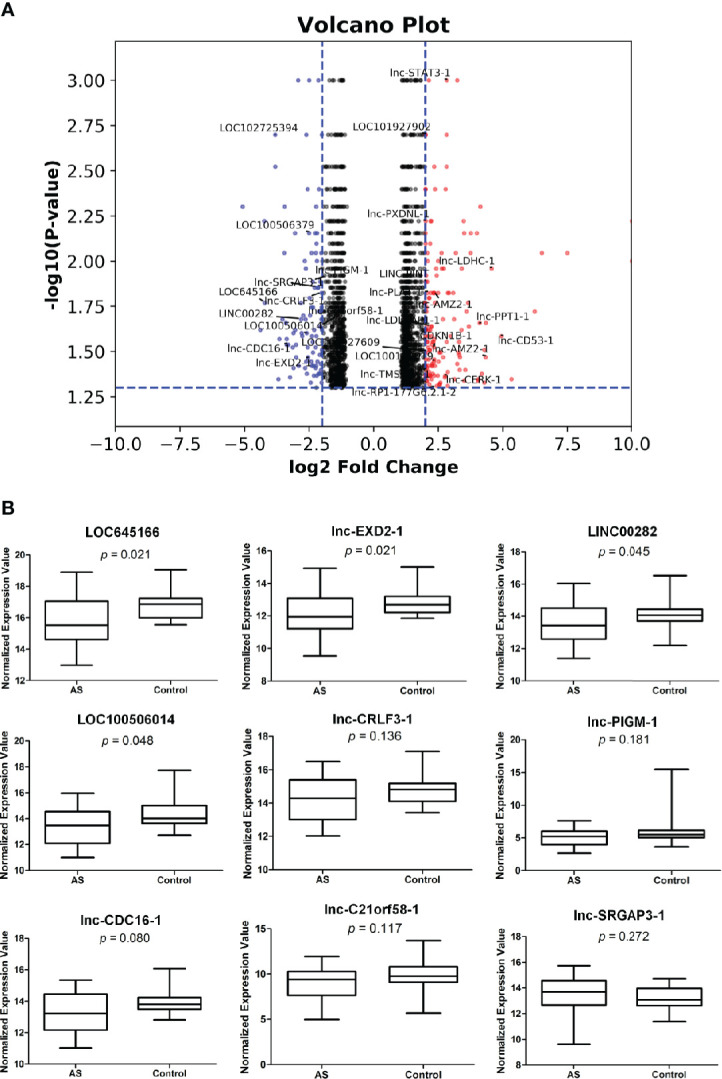
The differential expressions of lncRNAs in T cells of ankylosing spondylitis (AS) patients and healthy controls. **(A)** The volcano plot illustrating RNAs differential expression profiles with p value < 0.05 and > log2 fold change in T cells of the two groups. The two vertical blue lines represent a 2 log2 fold change upregulation and downregulation, respectively. The horizontal blue line refers to a p value of 0.05 (−log_10_ value). Compared with the healthy controls, the red and blue dots represent upregulated and downregulated RNA expressions more than 2 log2 fold change, respectively, in T cells of AS patients. LncRNAs in the red and blue spots are marked. **(B)** Quantitative real-time PCR. The lncRNAs marked in blue spots of **(A)** in T cells of two groups were analyzed by quantitative real-time PCR. Relative expression levels of lncRNA were determined by the following equation: (40 − threshold cycle [Ct] after adjustment by the expression of 18S rRNA). T cells isolated from 30 AS patients and 20 healthy controls were used in assays.

We have systematically analyzed the extensive differences in the expression levels of RNAs between T cells isolated from AS patients and healthy controls by using microarrays. The differential expression levels in total RNAs of T cells between two groups analyzed by microarray assay with p value < 0.05 and > log2 fold change are shown in **Supplementary Figure 1**. Compared with the healthy controls, the differential expression levels of RNAs up-regulated and down-regulated with p value < 0.05 and > log2 fold change are plotted in **Figure 1A**, respectively. 17 and 9 lncRNAs in T cells of AS patients were up-regulated and down-regulated more than 2 log2 fold change with p value < 0.05, respectively, are marked in **Figure 1A**. Then, we carried out the quantitative RT-PCR to analyze the down-regulated group and confirmed that four lncRNAs, LOC100506014, LOC645166, lncEXD2-1, and LINC00282, of them were significantly decreased in T cells of AS patients (**Figure 1B**).

### Overexpression of LOC645166 in Jurkat Cells Down-Regulates the Expression of *IL23p19* and Suppresses the JAK2/STAT3 Signaling in Response to Treatment With PMA or With Anti-CD3/CD28 Antibodies

**Figure 2:**
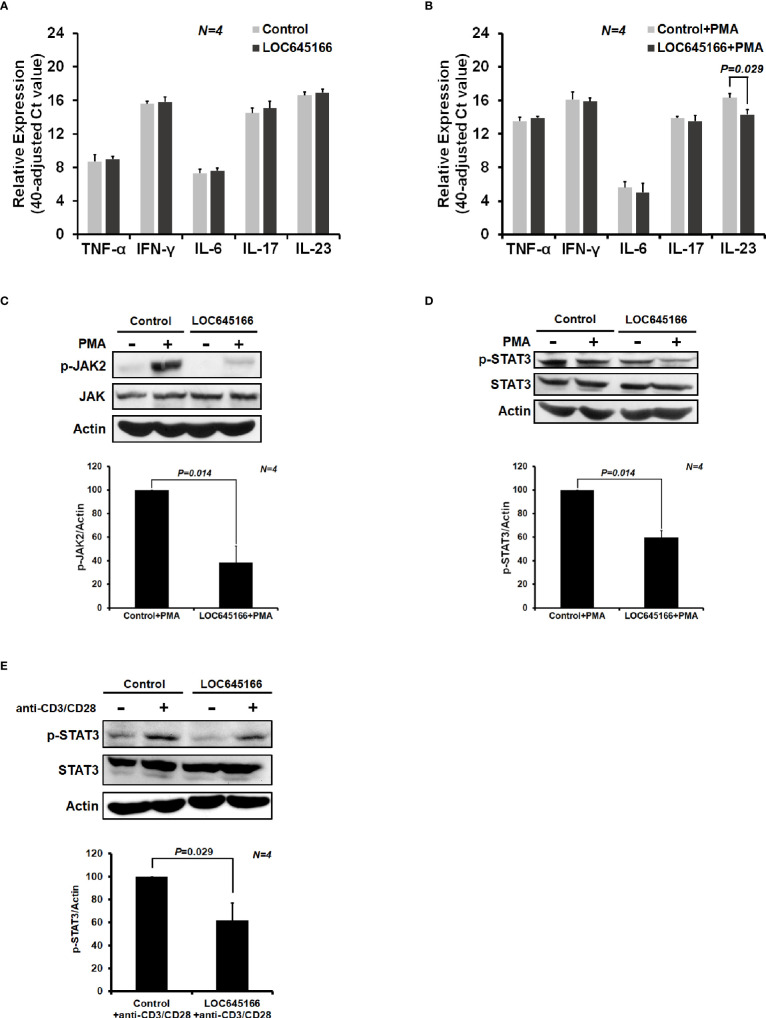
The effect of LOC645166 on the cytokine expression and on the JAK2/STAT3 signaling in response to treatment with phorbol 12-myristate 13-acetate (PMA) or with anti-CD3/CD28 antibodies. The effect of LOC645166 overexpressed in Jurkat cells on the expression of proinflammatory cytokines was carried out in the absence **(A)** and presence **(B)** of PMA treatment. The effect of LOC645166 ovexpressed in Jurkat cells on the suppression of JAK2/STAT3 signaling was performed in response to treatment with PMA **(C, D)** or with anti-CD3/CD28 antibodies **(E)**.

In order to characterize whether LOC645166 is involved in regulation of inflammatory reactions, LOC645166 was cloned and overexpressed in Jurkat cells. The mRNA levels of proinflammatory cytokines were analyzed by using quantitative RT-PCR after the LOC645166-overexpressed cells were treated with PMA. PMA alone can stimulate the NF-κB signaling (46). **Figure 2A** shows that overexpression of LOC645166 has no effect on the mRNA expression levels of proinflammatory cytokines, TNF-α, INF-γ, IL6, IL17, and IL23. However, when the LOC645166-overexpressed Jurkat cells were treated with PMA, the expression of IL23 was suppressed (**Figure 2B**). In addition, we asked whether overexpression of LOC645166 affects the IL23 signaling in response to treatment with PMA or with anti-CD3/CD28 antibodies. IL23 binds to its receptor and induces the dimerization of its receptor, in turn initializing JAK2/STAT3 signaling. The JAK2/STAT3 signaling is activated by phosphorylation. Therefore, we analyzed whether overexpression of LOC645166 in Jurkat cells can suppress the levels of phospho-JAK2 and phospho-STAT3 in response to treatment with PMA or with anti-CD3/CD28 antibodies. Overexpression of LOC645166 in Jurkat cells can suppress the phosphorylation of JAK2 and STAT3 in response to treatment with PMA (**Figures 2C, D**) or with anti-CD3/CD28 antibodies (**Figure 2E**), indicating that the signaling of IL23 was reduced after overexpression of LOC645166. In addition, suppression of STAT3 activation by LOC645166 overexpression was observed when T cells isolated from the healthy control or AS patient and MOLT4 cells (human T lymphoblast) were treated with anti-CD3/CD28 antibodies (**Supplementary Figure 2**) and with PMA (**Supplementary Figure 3**), respectively.

### LOC645166 Prefers to Interact With K63-Linkage Polyubiquitin Chains

Many lncRNAs regulate cellular functions through the effects on their associated proteins (17). Therefore, we prepared the biotinylated LOC645166 to carry out RNA pull-down assay. The proteins extracted from the nucleus of Jurkat cells were incubated with the biotin-conjugated LOC645166, followed by pull-down with streptavidin-conjugated beads. The precipitated proteins were subjected to analysis by shotgun proteomics. 64 proteins are only precipitated by biotinylated LOC645166 and shown in the **Supplementary Figure 4**.

**Figure 3:**
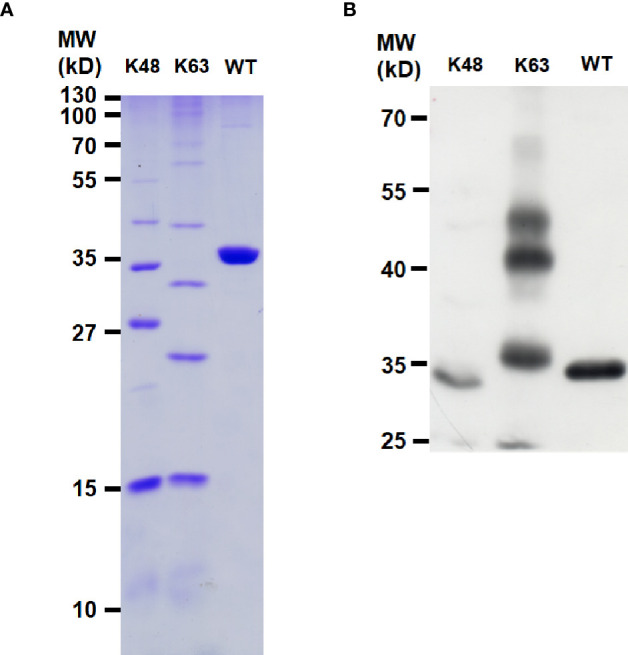
Characterization of LOC645166-binding preference to polyubiquitin chains. **(A)** SDS-PAGE analysis of the K48-, K63-, and linear-linked polyubiquitin chains. An aliquot (5 µg) of polyubiquitin chain (K48-, K63-, or linear-linked form) was analyzed by the SDS-PAGE (15%) and stained by Coomassie Brilliant Blue. **(B)** RNA pull-down assay to analyze the interaction between LOC645166 and the K48-, K63- or linear-linked polyubiquitin chains. K63- and K48-linked polyubiquitin chains consist of the complexes from monoubiquitin to heptaubiquitin and covalently linked by the isopeptide bonds through K63 and K48 of ubiquitin to the C-terminal glycine of another ubiquitin, respectively. The linear-linked polyubiquitin chain is a tetraubiquitin. An aliquot (1 µg) of polyubiquitin chain (K48-, K63-, or linear-linked form) was used for LOC645166 pull-down assay. The LOC645166-precipitated product was analyzed by western blotting, probed for ubiquitin by using anti-ubiquitin monoclonal antibody.

Polyubiquitin-C encoded by *UBC* gene in human is a linear polymer of nonaubiquitin (47). LOC645166 associated with polyubiquitin-C attracted our attention since K11-, K48- K63-, and linear-linked polyubiquitin chains are involved in NF-κB activation (20). These types of polyubiquitin chains implicated in NF-κB activation are synthesized by post-translational modifications in response to stimulation by the proinflammatory cytokines or ligands of TLR. In addition to polyubiquitin-C, we asked whether LOC645166 also binds to other types of polyubiquitin chains and which type of polyubiquitin chains is preferred to associate with LOC645166. The same amounts of K48-, K63-, and linear-linked polyubiquitins, as judged by SDS-PAGE stained with Coomassie Brilliant Blue (**Figure 3A**), were carried out for LOC645166 pull-down assay. **Figure 3B** shows that LOC645166 prefers binding to K63-linked polyubiquitin chains.

### Overexpression of LOC645166 Reduces the IKK Complex Recruited to K63-Linked Polyubiquitin Chains

**Figure 4:**
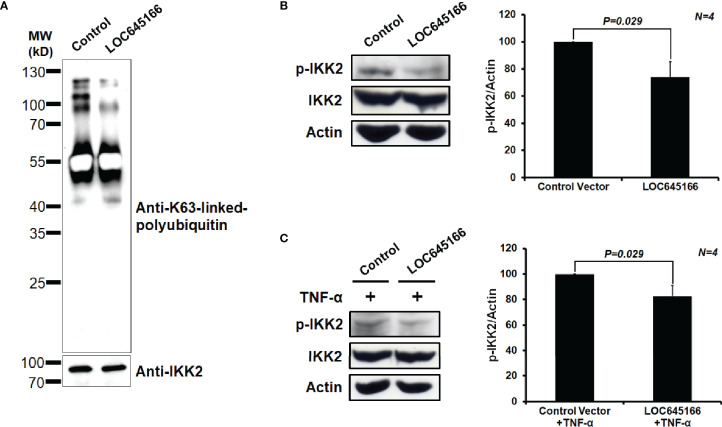
The effect of LOC645166 overexpressed in Jurkat cells on IKK2 activation in response to TNF-α treatment. **(A)** The effect of LOC645166 on recruitment of the IKK complex to the K63-linked polyubiquitin chains. The co-immunoprecipitated proteins by anti-IKK2 monoclonal antibody were analyzed by western blotting, probed for K63-linked polyubiquitin chains by using anti-K63-linkage specific polyubiquitin monoclonal antibody or IKK2 by anti-IKK2 monoclonal antibody. **(B)** The effect of LOC645166 overexpression on the IKK2 phosphorylation. The ratios of p-IKK2/actin averaged from four independent experiments are plotted. The ratios of p-IKK2/actin obtained from cells transfected with control vector are set to 100%. **(C)** The effect of LOC645166 overexpression on the IKK2 phosphorylation in response to treatment with TNF-α. The ratios of p-IKK2/actin averaged from four independent experiments are plotted. The ratios of p-IKK2/actin obtained from cells transfected with control vector plus TNF-α treatment are set to 100%.

LOC645166 prefers binding to the K63-linked polyubiquitin chains, reflecting that it may regulate the NF-κB activation through blocking recruitment of the IKK complex to the polyubiquitin chains and attenuate phosphorylation of IKK2. Thus, we analyzed whether overexpression of LOC645166 in Jurkat cells can diminish the IKK complex recruited to K63-linked polyubiquitin chains. **Figure 4A** shows that overexpression of LOC645166 results in a decrease of IKK2 recruited to K63-linked polyubiquitin chains in response to TNF-α treatment, as evidenced by the reduced levels of K63-linked polyubiquitin chains co-immunoprecipitated by anti-IKK2 monoclonal antibody. In addition, overexpression of LOC645166 can reduce the IKK2 phosphorylation in response to TNF-α treatment (**Figures 4B, C**), but no effect on IKK2 expression.

### Overexpression of LOC645166 Reduces the Phosphorylation of IκB and p50 Translocation From Cytosol to Nucleus

**Figure 5:**
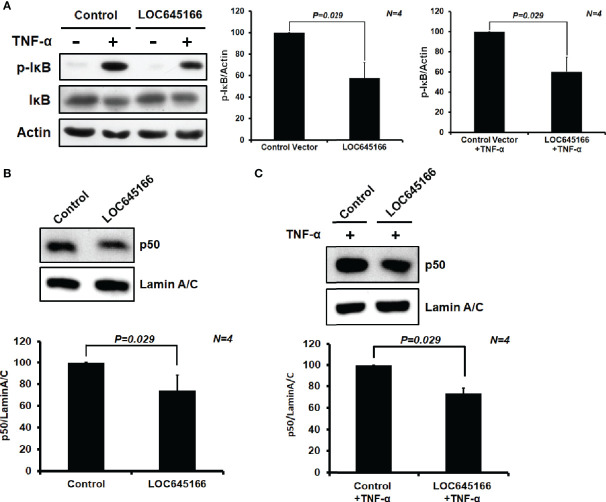
The effect of LOC645166 overexpressed in Jurkat cells on the NF-κB signaling. **(A)** The effect of LOC645166 overexpression on the IκB phosphorylation. The ratios of p-IκB/actin averaged from four independent experiments are plotted. The ratios of p-IκB/actin obtained from cells transfected with the control vector or the control vector plus TNF-α treatment are set to 100%. The effect of LOC645166 overexpression on translocation of p50 from cytosol to nucleus was carried out in the absence **(B)** or presence **(C)** of stimulation by TNF-α. The ratios of p50/lamin A/C averaged from four independent experiments are plotted. The ratios of p50/lamin A/C obtained from cells transfected with the control vector in the absence **(B)** and presence **(C)** of TNF-α treatment are set to 100%, respectively.

LOC645166 can attenuate the activation of IKK2 *via* blocking the IKK complex recruited to K63-linked polyubiquitin chains. Thus, we would like to ask whether overexpression of LOC645166 can suppress IκB phosphorylation. The LOC645166-overexpressed Jurkat cells were treated with TNF-α for 6 h and the levels of phospho-IκB were analyzed by western blotting. **Figure 5A** shows that overexpression of LOC645166 can suppress the phosphorylation of IκB, suggesting that the NF-κB activation is reduced. Indeed, overexpression of LOC645166 can attenuate p50, one of the NF-kB components, translocation from cytosol to nucleus (**Figures 5B, C**).

## Discussion

LOC645166 is a long noncoding RNA. We found that the levels of LOC645166 are significantly reduced in T cells of AS patients by microarray analysis and quantitative RT-PCR (**Supplementary Figure 1** and **Figures 1A, B**). Overexpression of LOC645166 can suppress the *IL23p19* expression (**Figure 2B**) and JAK2/STAT-3 signaling, induced by IL23, of Jurkat cells in response to treatment with PMA (**Figures 2C, D**). IL23, a heterodimer consisting of IL12p40 and IL23p19, is a key cytokine for growth and activation of Th-17 cells that characteristically produce IL6, IL17, and TNF-α, and drive autoimmune inflammatory (48). The *IL23p19* gene expression is highly regulated NF-κB signaling mediated by TLRs. Three putative NF-κB binding sites are located within the promoter region of *IL23p19* gene and two of them are efficiently associated with c-Rel, a member of NF-κB family (48). Mutation at either of two c-Rel binding sites entirely disrupts the promoter activity of *IL23p19* gene. The effect of LOC645166 on the *IL23p19* gene expression suggests that it may regulate the NF-κB activity.

Many lncRNAs carry out their activities to regulate cell functions *via* the effect on their associated proteins. 64 proteins precipitated by LOC645166 were identified by using RNA pull-down assay plus a proteomic approach in our study (**Supplementary Figure 4**). Among them, polyubiquitin-C is a special LOC645166-binding protein. Polyubiquitin-C is encoded by *UBC* gene in human and is a linear nonaubiquitin. In ployubiquitin-C, the C-terminal Gly of ubiquitin is repeatedly and covalently linked to N-terminal Met of another ubiquitin. Polyubiquitin-C is one of the major sources of cellular ubiquitin under stress conditions and is essential for fetal liver cell proliferation in mouse (47). Knockout of *UBC* gene results in mouse embryonic fibroblasts displaying reduced cell growth, up-regulated apoptosis, and delayed cell cycle progression (47). In addition to the linear form of polyubiquitin-C produced by translation from its mRNA, several Lys-linked and linear-linked forms of polyubiquitin chains are also synthesized *via* an intricately post-translational modifications in cells (49). Recent studies have revealed that the K11-, K63-, or linear-linked polyubiquitin chains are also involved in NF-κB activation (20). Three hydrophobic Ile44, Ile36, and Phe4 patches as well as TEK-box are distributed on the surface of ubiquitin (49). Structural characterization reveals that the different linkages in the polymerized ubiquitin chain create the distinct surface at the interface for interaction with their neighboring moieties, leading to ubiquitin chains adopting compact or open conformations (49). In compact conformations, ubiquitin moieties tightly pack each other at their interface contacted with different hydrophobic patches in different Lys-linked diubiquitin, resulting in the side-chains of different residues distributed on the surface of ubiquitin chains. The K48-, K11-, and K6-linked polyubiquitin chains adopt the compact conformations. In open conformations, the linkage sites do not display any interface for interaction with ubiquitin moieties, exhibiting the high conformational freedom of ubiquitin chains. The K63- and linear-linked polyubiquitin chains adopt the open conformations (47).

Due to the different side-chains distributed on the surface of ubiquitin chains, the different Lys- or linear-linked polyubiquitin chains will attract distinct binding molecules. The RNA pull-down assay demonstrated that LOC645166 prefers binding to the K63-linked more than to K48- and linear-linked polyubiquitin chains (**Figure 3B**). The binding characteristic of LOC645166 allows it to block recruitment of the IKK complex to K63-linked polyubiquitin chains, where the dimerized IKK complex can trigger the IKK2 auto-phosphorylation by each other and activate IKK2 kinase activity. Indeed, overexpression of LOC645166 in Jurkat cells suppresses the levels of K63-linked polyubiquitin chains co-immunoprecipitated by anti-IKK2 antibody (**Figure 4A**). This consequence reflects that the activation of IKK2 by auto-transphosphorylation mediated by TLR was suppressed by LOC645166 *via* association with K63-linked polyubiquitin chains (**Figures 4B, C**), in turn leading to down-regulation of IκB phosphorylation by the activated IKK2 (**Figure 5A**). The stable IκB binds to NF-κB, allowing the NF-κB/IκB complex retained in the cytosol. Translocation of p50 from cytosol to nucleus is attenuated (**Figures 5B, C**). The *IL23p19* gene expression and the IL23 signaling mediated by TLRs are suppressed in LOC645166-overexpressed Jurkat cells (**Figure 2B**). Our results revealed that down-regulation of LOC645166 expression in T cells allows AS patients to enhance their sensitivities to activate the NF-κB signaling in response to stimulation by pro-inflammatory cytokines or by TLR ligands. Up-regulation of LOC645166 expression in T cells is capable of suppressing the sensitivity for NF-κB activation, providing the potential therapeutic target for AS treatment.

## Data Availability Statement

The original contributions presented in the study are included in the article/**Supplementary Material**. Further inquiries can be directed to the corresponding authors.

## Ethics Statement

The studies involving human participants were reviewed and approved by The Institutional Review Board and Ethics Committee (IRBEC) of Buddhist Dalin Tzu-Chi General Hospital. The patients/participants provided their written informed consent to participate in this study.

## Author Contributions

H-CY, K-YH, and H-YH contributed to method design, experiments, and data analysis. M-CL and S-QL contributed to data analysis. H-CY, N-SL, and H-BH contributed to method design, data analysis, and manuscript writing. All authors contributed to the article and approved the submitted version.

## Funding

This work was supported by grants from the Ministry of Science and Technology, ROC (MOST 108-2314-B-303-015-MY2), from the Buddhist DaLin Tzu-Chi Hospital [DTCRD 104(2)-I-01-03; DTCRD 105-E-16], and from the Buddhist Tzu Chi Medical Foundation [TCMF-SP108-02(108)].

## Conflict of Interest

The authors declare that the research was conducted in the absence of any commercial or financial relationships that could be construed as a potential conflict of interest.
