# Supplementary figures and images for: Down-Regulation of LOC645166 in T Cells of Ankylosing Spondylitis Patients Promotes the NF-κB Signaling via Decreasingly Blocking Recruitment of the IKK Complex to K63-Linked Polyubiquitin Chains
Source: Front Immunol. 2021 Feb 25;12:591706. doi: 10.3389/fimmu.2021.591706 (PMC7946993; doi:10.3389/fimmu.2021.591706)

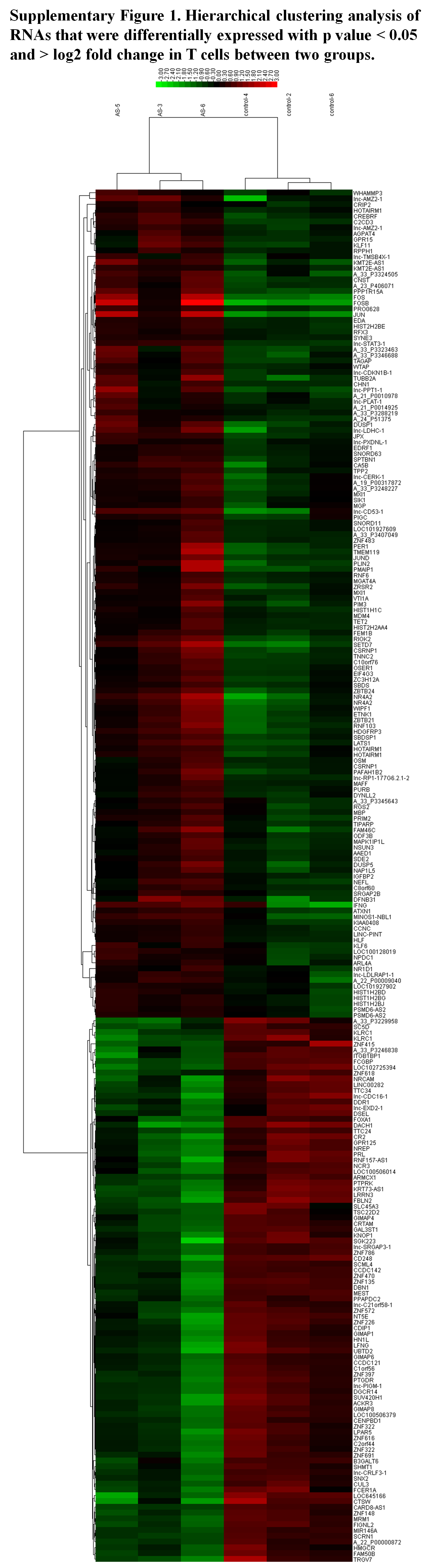

Supplement: Supplementary file 1 [file Image_1.tif]

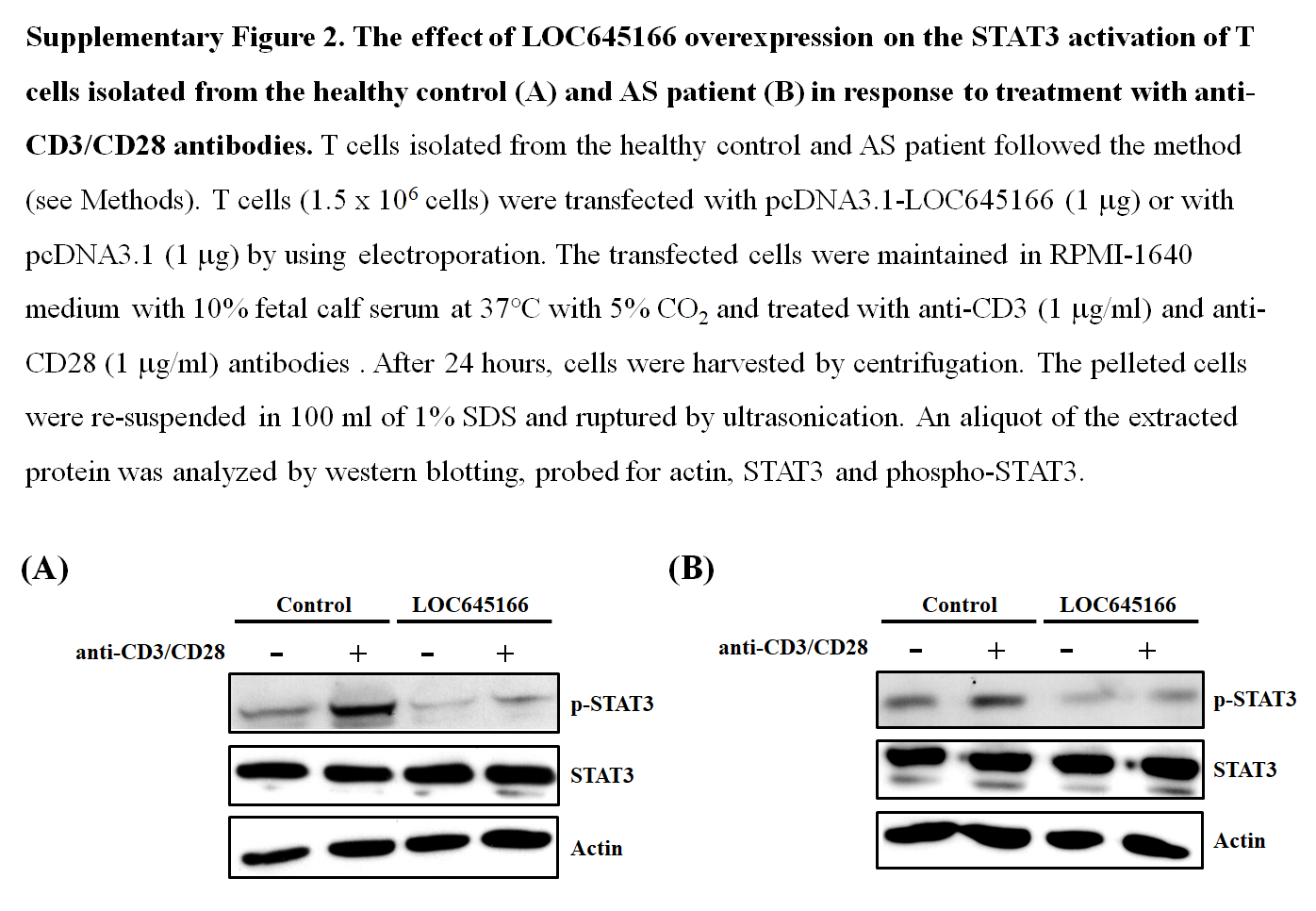

Supplement: Supplementary file 2 [file Image_2.tif]

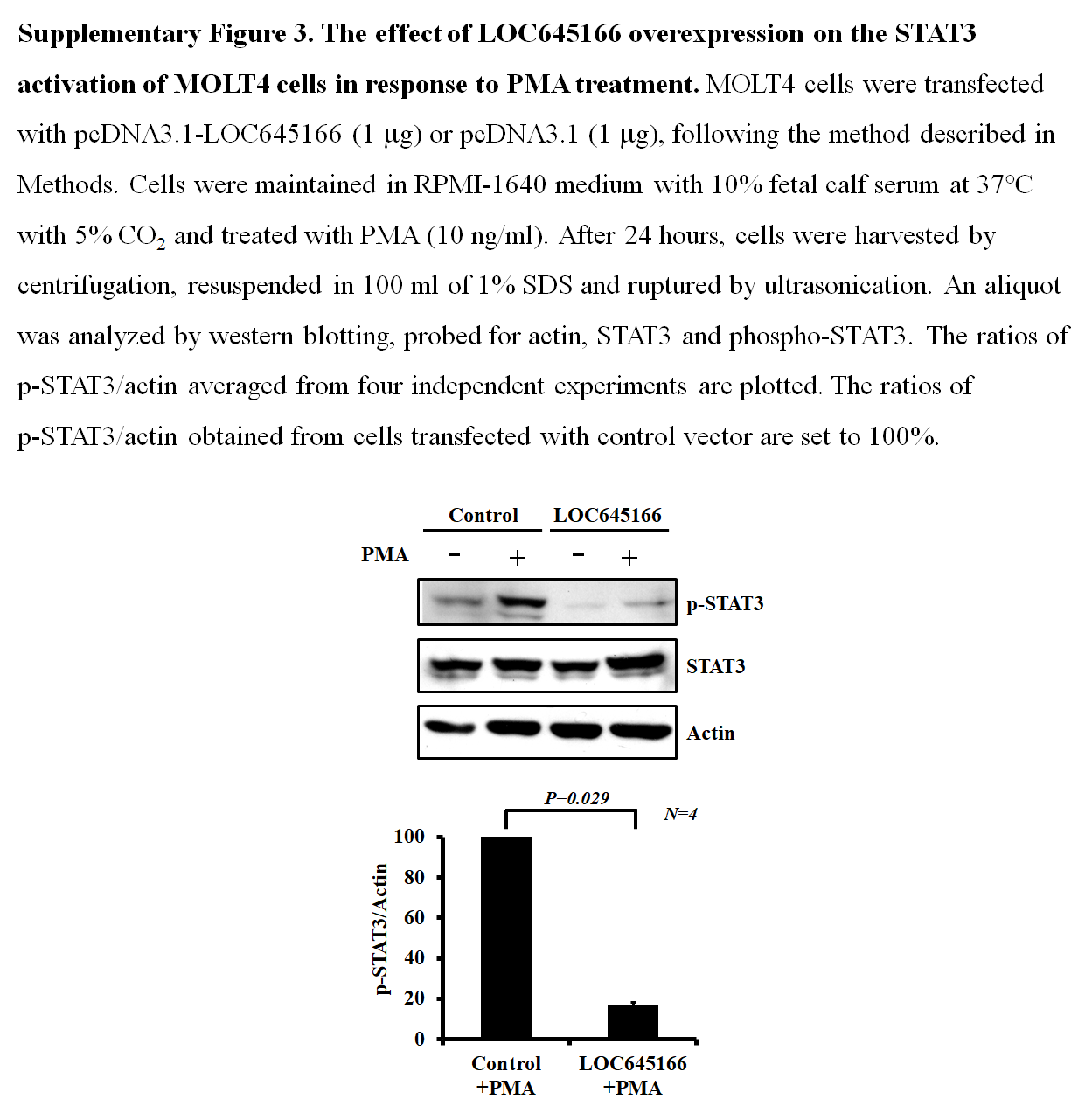

Supplement: Supplementary file 3 [file Image_3.tif]

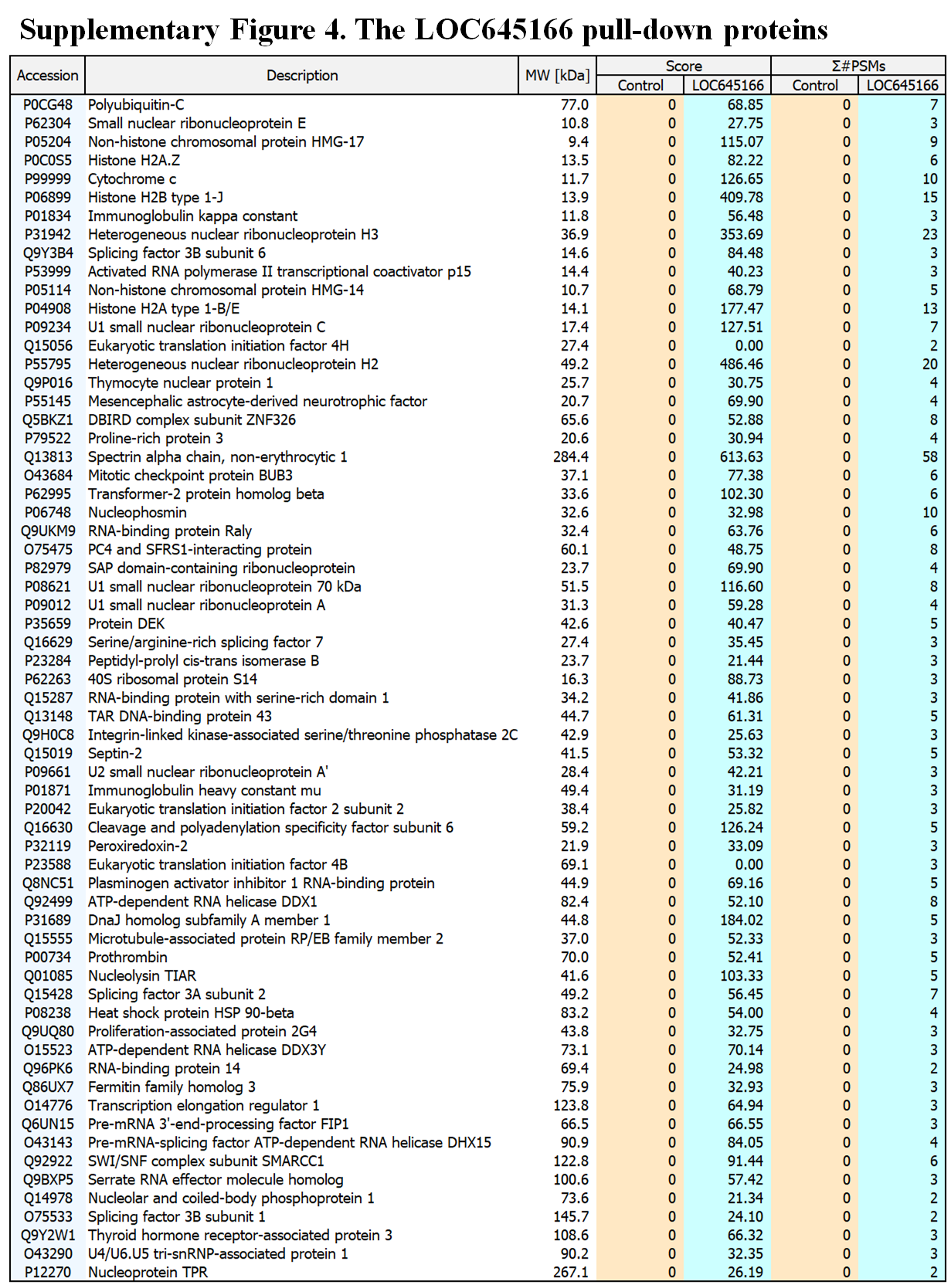

Supplement: Supplementary file 4 [file Image_4.tif]
