# Supplementary material for: Down-Regulation of LOC645166 in T Cells of Ankylosing Spondylitis Patients Promotes the NF-κB Signaling via Decreasingly Blocking Recruitment of the IKK Complex to K63-Linked Polyubiquitin Chains
Source: Front Immunol. 2021 Feb 25;12:591706. doi: 10.3389/fimmu.2021.591706 (PMC7946993; doi:10.3389/fimmu.2021.591706)
Supplement: Supplementary file 5 [file Table_1.docx]

**Supplementary Table I. The primers used in quantitative real-time PCR.**

| **Long noncoding RNA** | **Primers (5’-forward/3’-reverse)** |
| --- | --- |
| LOC645166 | GCAGAGATGAATGTGCCTGGA/CAGTGAGTAATAATCCCTAACC |
| Lnc-EXD2 | GTGTTACCCTCCAGCACTGT/GGGTGAATGAATGGATACAGC |
| LINC-00282 | GCTACATCGGGCATCATTCTA/AGTCCTTGGGTAGCTCTCAG |
| LOC100506014 | GGGAGGAGATAACATGAGGG/TGCAGCTGACGAGTCAGGG |
| Lnc-CRLF3-1 | TTCACAGGAGTGCCTTAAACC/CAGGAGACAGAGGTTGCAGT |
| Lnc-PIGM-1 | GTGAAATGGGAATGATGACACA/GGTTCAAGTTTACAGTGCCAG |
| Lnc-CDC16-1 | ACAGCTGTCCTAGCGCCATG/ATGGCAGCAGATCCTCCCTA |
| Lnc-C21orf58-1 | CTATGGCAGACAGTGGTGGA/TACAGAGATGAGCCATCACTG |
| Lnc-SRGAP3-1 | GTTTAGCAATTCTCCTGCCTC/TCTCCTGGATCACGATTCACA |
